# 

**Published:** 1847-08

**Authors:** 


### NOTICE TO READERS AND CORRESPONDENTS.

We have received original communications from Drs. McLean, J. E. McGirr, and A. W. Benton.

The following works have been received:

Observations on Aneurism and its Treatment by Compression. By O'Bryen Bellingham, M.D., Edin., Fellow of and Professor in the School of the Royal College of Surgeons in Ireland, etc., etc. London: John Churchill, Princes Street, Soho. 1847. pp. 181, 12 mo. (Arrived too late to be noticed.)

Hand Book of Human Anatomy, General, Special, and Topographical. Translated from the original German of Dr. Alfred Von Behr, and adapted to the use of the English Student, by John Birkett, Fellow of the Royal College of Surgeons of England, and Demonstrator of Anatomy at Gray's Hospital. Phila.: Lindsay and Blakiston. 1847. pp. 487, 12mo. (From the Publishers, for sale by Joseph Keen, Jr., Chicago.)

A Treatise on the Practice of Medicine. By George B. Wood, M.D., Professor of Materia Medica and Pharmacy in the University of Pennsylvania, one of the Physicians to the Pennsylvania Hospital, one of the Authors of the Dispensary of the United States of America. In two vols. Philad.: Grigg, Elliot, & Co. 1847. vol. i, pp. 791, vol. ii, pp. 840, 8vo. (From the Publishers. For sale by A. H. & C. Burley, Chicago.)

Proceedings of National Medical Convention, held in New York May, 1846, and in Philadelphia May, 1847.

Some Account of the Letheon; or, Who is the Discoverer? By Edward Warren. Boston, 1847. Pamphlet.

Circular—Patent Letheon. Pamphlet.

Triumphs of "Young Physic," or Chrono-Thermal Facts. By Wm. Turner, Esq., A.M., M.D. New York. Pamphlet.

Annual Circular of the Medical Institution of Geneva College, Session of 1847.

Catalogue and Circular of the Albany Medical College, 1847.

Annual Announcement of Rush Medical College, Chicago, Ill., 1847.

Annual Announcement of the Medical Department of the St. Louis University, 1847.

The Harmony of Creation. An address delivered before the Medical Department of the West[illegible] Reserve College, Cleveland. By Rev. Elijah Burrows.

Catalogue of the Medical Department of the University of the State of Missouri, 1847.

Catalogue of the Trustees, Officers, and Students of the Indiana Medical College, Session of 1846-'47.

A Practical Treatise on the Diseases of Children. By D. Francis Condie, M.D., Secretary of the College of Physicians; Member of the American Philosophical Society; Honorary Member of the Philadelphia Medical Society, etc. Second Edition, Revised and Augmented. Philadelphia.: Lea & Blanchard. 1847. 8vo, pp. 657. (From the Publishers. For sale by Joseph Keen, Jr., Chicago.)

The Medical Students' Vade Mecum, or Manual of Examinations upon Anatomy, Physiology, Chemistry, Materia Medica, Surgery, Obstetrics, Practice of Medicine, (including Physical Diagnosis and Diseases of the Skin,) and Poisons. Second Edition, Revised and Enlarged. By George Mendenhall, M.D., Lecturer on Pathology in the Medical Institute of Cincinnati; Member of the Philadelphia Medical Society. &c., &c. Philadelphia: Lindsay and Blakiston. 1847. pp. 574. (From the Publishers.)

Medical Botany; or Descriptions of the more important Plants used in Medicine, with their History, Properties, and Mode of Administration. By R. Eaglesfield Griffith, M.D., Member of the American Philosophical Society; of the Academy of Natural Sciences of Philadelphia, etc., etc.; with upwards of three hundred illustrations. Philadelphia: Lea & Blanchard. 1847. 8vo., pp. 704. (From the Publishers, and for sale by Joseph Keen, Jr., Chicago.)

A Practical Treatise on Inflammation, Ulceration, and Induration of the Neck of the Uterus, with Remarks on Leucorrhœ and Prolapsus Uteri, as Symptoms of Uterine Disease. By James Henry Bennet, M.D., Licentiate of the Royal College of Physicians, &c., &c. Philadelphia: Lea & Blanchard. 1847. pp. 146. (From the Publishers, by Joseph Keen, Chicago.)

Poisonous Effects of Quinine. By W. O. Baldwin, M.D., of Montgomery, Alabama. Pamphlet.

We have received in exchange the following periodicals:

Ranking's Half-Yearly Abstract of the Medical Sciences.
The American Journal of the Medical Sciences.
The Medico-Chirurgical Review and Journal of Practical Medicine.
Braithwaite's Retrospect of Practical Medicine and Surgery. Part xiv.
New York Journal of Medicine, etc., New York.
The Annalist, a Record of Practical Medicine, New York.
New York Medical and Surgical Reporter, New York.
The Medical Examiner, etc., Philadelphia.
The Medical News end Library, Philadelphia.
The Western Journal of Medicine and Surgery, Louisville, Ky.
The Western Lancet and Medical Library, Lexington, Ky.
The Southern Medical and Surgical Journal, Augusta, Georgia.
The Boston Medical and Surgical Journal, Boston, Mass.
The Missouri Medical and Surgical Journal, St. Louis, Missouri.
The St. Louis Medical and Surgical Journal, St. Louis, Missouri.
The Practical Educator and Journal of Health, Boston.
Stockton and Co.'s Dental Intelligencer, Philadelphia.
Dublin Medical Gazette.

---

### CONTRIBUTORS TO THE ILL. AND IND. MED. AND SURG. JOUR.

S. G. Armor, M. D., Rockford, Ill.
A. H. Howland, M. D., Ottawa, Ill.
A. G. Henry, M. D., Pekin, Ill.
Daniel Stahl, M. D., Quincy, Ill.
Edward Mead, M. D., Geneva, Ill.
H. S. Huber, M. D., Chicago, Ill.
David Prince, M. D., Jacksonville, Ill.
P. A. Allaire, M. D., Aurora, Ill.
J. F. Henry, M. D., Burlington, Iowa.
Jno. McLean, M. D., Jackson, Mich.
Edward Lewis, M. D., Jackson, Mich.
Ira C. Bachus, M. D., Jackson, Mich.
J. G. Conwell, M.D., Spring Arbor, Mich.
S. B. Thayer, M. D., Battle Creek, Mich.
E. Deming, M. D., Lafayette, Ind.
G. N. Fitch, M. D., Logansport, Ind.
G. W. Clippinger, M.D., Terre Haute, Ia.
Elias Fisher, M. D. Waynesville, Ohio.

